# Tension-Compression Loading with Chemical Stimulation Results in Additive Increases to Functional Properties of Anatomic Meniscal Constructs

**DOI:** 10.1371/journal.pone.0027857

**Published:** 2011-11-16

**Authors:** Daniel J. Huey, Kyriacos A. Athanasiou

**Affiliations:** Department of Biomedical Engineering, University of California Davis, Davis, California, United States of America; University of Pittsburgh, United States of America

## Abstract

**Objective:**

This study aimed to improve the functional properties of anatomically-shaped meniscus constructs through simultaneous tension and compression mechanical stimulation in conjunction with chemical stimulation.

**Methods:**

Scaffoldless meniscal constructs were subjected to simultaneous tension and compressive stimulation and chemical stimulation. The temporal aspect of mechanical loadingwas studied by employing two separate five day stimulation periods. Chemical stimulation consisted of the application of a catabolic GAG-depleting enzyme, chondroitinase ABC (C-ABC), and an anabolic growth factor, TGF-β1. Mechanical and chemical stimulation combinations were studied through a full-factorial experimental design and assessed for histological, biochemical, and biomechanical properties following 4 wks of culture.

**Results:**

Mechanical loading applied from days 10–14 resulted in significant increases in compressive, tensile, and biochemical properties of meniscal constructs. When mechanical and chemical stimuliwere combined significant additive increases in collagen per wet weight (4-fold), compressive instantaneous (3-fold) and relaxation (2-fold) moduli, and tensile moduli in the circumferential (4-fold) and radial (6-fold) directions were obtained.

**Conclusions:**

This study demonstrates that a stimulation regimen of simultaneous tension and compression mechanical stimulation, C-ABC, and TGF-β1 is able to create anatomic meniscus constructs replicating the compressive mechanical properties, and collagen and GAG content of native tissue. In addition, this study significantly advances meniscus tissue engineering by being the first to apply simultaneous tension and compression mechanical stimulation and observe enhancement of tensile and compressive properties following mechanical stimulation.

## Introduction

The knee joint is a complex system of tissues that each lends unique contributions to proper joint functionality. The fibrocartilaginous meniscus provides the important function of protecting the articular cartilage from receiving the full stresses transmitted through the knee joint [1], [2]. The ability to perform this function is due to the unique geometric, biochemical, and biomechanical properties of the meniscus [3]. Due its load bearing nature, the meniscus, particularly the inner region, is a commonly injured tissue. Following injury, the lack of vascularity and the inability for intrinsic repair of the inner portion of the meniscus ensures a functional healing response does not ensue [3], [4]. This places the underlying articular cartilage under non-physiologic loading causing it to enter an osteoarthritic pathway [1], [2]. Thus, it is critical to regain meniscus structure and function following injury. Unfortunately, the current standard of treatment for meniscal injuries is partial meniscectomy, which relieves the immediate discomfort of meniscal tearing but does nothing to prevent the osteoarthritic sequela [5]. While acellular replacements are currently employed for treatment of inner portion meniscal tearing [6], the use of a living biological tissue would likely be preferred due to concerns of shrinkage following implantation and potential enhancement of implant to tissue integration [6]. The lack of a technique able to replace damaged meniscal tissue through replication of both geometric and functional properties and the scarcity of donor tissue for meniscal allografting both motivate the desire to tissue engineer living inner-meniscus tissue.

Recently, a scaffold-free method of construct formation, the self-assembly, process has been shown to generate cartilaginous and fibrocartilaginous tissue with compressive properties approaching those of native tissue [7]–[11]. This process allows for the creation of geometrically complex tissue constructs by seeding cells into an appropriately shaped, non-adherent agarose well. Guided by the Differential Adhesion Hypothesis, the cells attempt to limit their free energy by binding to each other via N-cadherin connections [10]–[12]. This scaffold-free method of forming a meniscal construct avoids drawbacks associated with scaffold usage including stress shielding, biocompatibility of the material and its degradation products, and fibroblastic changes in cell morphology due to adhesion [13], [14].

Researchers have investigated a myriad of stimuli aimed at enhancing functional properties of engineered tissue [11], [15]–[21]. TGF-β1 is one of the most commonly applied growth factors for cartilage engineering and has been shown to enhance the biochemical and biomechanical properties of cartilage constructs [22]–[26]. The application of chondroitinase ABC (C-ABC) to cartilage constructs is less well studied but has been shown to be effective at increasing collagen per wet weight and tensile properties of engineered cartilage and native tissue [11], [18], [27]–[29]. The mechanism for these increases has not yet been elucidated but current hypotheses revolve around enhancement of the collagen matrix via matrix-matrix, cell-matrix, or cell-cell interactions that would be sterically hindered prior to C-ABC treatment, release of bound growth factors, or purely a biophysical effect [11], [18], [27]–[29]. The particular combination of C-ABC and TGF-β1 applied in this study was chosen based on a previous study which examined intermittent and continuous treatment of TGF-β1 combined with C-ABC treatment after 1 wk or 2 wks of culture on self-assembled meniscal constructs [11]. This study found that the continuous application of TGF-β1 coupled with C-ABC application after 1 wk culture resulted in synergistic enhancement of construct biochemical and biomechanical properties. The present study will build upon these results by applying the aforementioned temporally-coordinated chemical stimulation regimen in conjunction with mechanical stimulation.

While the effects of deformational mechanical stimulation on engineered articular cartilage have been well studied [21], [30]–[37], there is a dearth of studies on the effects of mechanical stimulation on meniscal constructs [19], [38]–[41]. Previous work applying either dynamic compression or tension to meniscus constructs observed decreases in proline and sulfate incorporation [38], increased proliferation [38], improvements in either proline and sulfate incorporation [39], [40], or increases in collagen and GAG content and stiffness [19]. In addition a recent study applied compression stimulation to an anatomically-shaped meniscus construct and observed significant enhancement of collagen and GAG per wet weight and compressive properties. Due to the lack of studies examining meniscus mechanical stimulation and the dual cartilaginous and fibrous nature of meniscal constructs, the use of compressive and tensile stimulation to engineer cartilage and tendon, respectively, can provide guidance. Compression stimulation has successfully been employed to enhance GAG and collagen synthesis and the compressive properties of articular cartilage constructs [21], [31], [36], [37]. Tensile stimulation of engineered tendon can increase tensile properties up to 3-fold [42], [43]. These accounts of successful application of mechanical stimulation to other mechanically functional tissues provide motivation to elucidate the effects of compressive and tensile stimulation on meniscal constructs.

As meniscal tissue is subjected to both tensile and compressive stimulation *in vivo*, it would be ideal to apply both of these in concert to developing meniscus constructs. The most elegant way to apply both of these forces would be to mimic the native meniscus loading condition. This requires a construct that possesses a curved-wedge profile to translate compressive loading to circumferential tensile loading and a ring shape to allow the generation of tensile forces within the constructs. Constructs possessing these characteristics have been created with the self-assembly process [8], [11] and a direct compression stimulator has been used to compress both meniscal and articular cartilage explants [44]. Thus, the present study will use this direct compression stimulator with custom fabricated compression platens matching the curvature of the meniscal constructs to apply simultaneous compression and circumferential tension loading.

Previous studies employing growth factors, C-ABC, well-confinement, and hydrostatic pressure to self-assembled constructs have found that the timing of stimulation has profound effects on resultant construct properties [11], [22], [28], [45], [46]. These studies indicate that the application of stimulation to self-assembled constructs is most effective when it occurs between 7 and 14 days following construct seeding. As such, the present study will attempt to identify an intervention window where self-assembled meniscal constructs are amenable to tension-compression stimulation.

The purpose of this present study is to examine the full factorial combinations of mechanical stimulation (at three levels) and chemical stimulation (at two levels). Chemical stimulation is defined as continuous application of TGF-β1 and a one time treatment of C-ABC after 1 wk. Mechanical stimulation is defined as simultaneous tensile and compressive loading during a 5 day period; and the three levels are application from days 10–14, application from days 17–21, or no application. We hypothesize that: 1) early mechanical stimulation will be more beneficial than later stimulation due to the level of construct “naiveté” and 2) additive increases to functional and biochemical properties will result from combined mechanical and chemical stimulation.

## Materials and Methods

### Cell Isolation

Femoral articular cartilage and medial and lateral menisci were sterilely isolated from knee joints of 1 wk old calves (Research 87). Following dicing of the tissue into ∼1 mm pieces, meniscal and articular cartilages were separately digested in 0.2% collagenase type II (Worthington) in cell culture medium. The medium formulation follows: Dulbecco's modified Eagle's medium (DMEM) (Invitrogen), 10% fetal bovine serum (FBS) (Benchmark), 1% non-essential amino acids (NEAA) (Invitrogen), 25 µg of l-ascorbic acid (Sigma) and 1% penicillin/streptomycin/fungizone (PSF) (Fisher Scientific). After 18 hrs of digestion, cells were isolated by multiple centrifugation and washing steps and filtration through a 70 µm mesh. Freezing media consisting of the media above with an additional 10% FBS and 10% DMSO (Fisher Scientific) was prepared and used to cryopreserve articular chondrocytes and meniscus cells. Freezing rate was controlled until −80°C was reached and then cells were placed in liquid nitrogen.

### Construct Seeding

The process employed for the creation of meniscus shaped self assembled constructs has been described previously [8], [11]. Briefly, positive dies in the shape of the rabbit meniscus were plunged into 2% molten agarose and after the agarose had set, the positive die was removed to create a negative mold. The wells were placed in chondrogenic medium which was allowed to infiltrate the well for 1 wk prior to seeding. Chondrogenic media formulation follows: DMEM (Invitrogen), 100 nM dexamethasone, 1% PSF (Fisher Scientific), 1% ITS+ (BD), 50 mg/mL ascorbate-2-phosphate, 40 mg/mL L-proline, and 100 mg/mL sodium pyruvate (Fisher Scientific). For construct seeding, articular chondrocytes and meniscus cells were thawed, combined in a 50∶50 ratio, and 20 million cell aliquots were placed into each well. Equal parts of articular chondrocytes and meniscus cells were chosen as previous studies have shown that this combination results in constructs most resembling meniscal tissue morphologically, biochemically, and biomechanically [7], [11]. Within 24 hours the cells had coalesced to form a tissue construct and by day 7 of culture were robust enough to remove from the confining agarose well. Every other day the culture medium was refreshed for the duration of the 4 wk study.

### Construct Stimulation

Two chemical agents, C-ABC (Sigma) and TGF-β1 (Peprotech), were applied following the regimen previously demonstrated to synergistically enhance constructs properties (denoted as CY) or neither agents were applied (denoted as CN) [11]. Specifically, TGF-β1 at 10 ng/mL was applied continuously throughout the entire duration and a one-time C-ABC treatment was applied for 4 hrs at 2 U/mL after 1 wk of culture.

**Figure 1:**
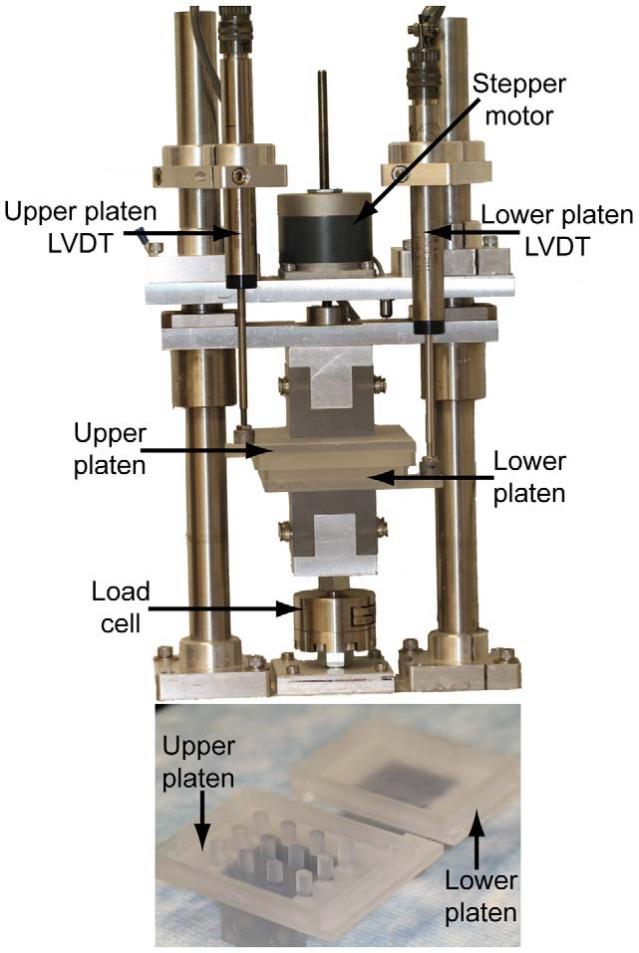
Tension-compression stimulator and platens. (Upper) Stimulation apparatus with relevant components labeled. (Lower) Rapid prototyped stimulation platens with upper platen possessing a curved surface to mate with the upper surface of meniscal constructs.

Mechanical stimulation was provided by the custom-built stepper motor driven, computer controlled direct compression stimulator shown in Figure 1 [44]. To obtain simultaneous compression and tension stimulation, platens were fabricated to match the curved surface and elliptical shape of the meniscal constructs. AutoCAD was used to create a 3D model of the platens and then this model was used in conjunction with stereolithography (Laser Reproductions) to create the functional tension-compression stimulation platens seen in Figure 1.

In this study mechanical stimulation was applied at three temporal levels: days 10–14 (denoted as D1 or early mechanical stimulation), days 17–21 (denoted as D2 or late mechanical stimulation) or never (denoted as DN). The axial strain percentage and application frequency were 10% and 1 Hz, respectively and stimulation was applied for 1 hr per day with 30 cycles of 1 minute dynamic stimulation and 1 minute of uncompressed rest. These specific mechanical stimulation parameters were selected based on prior studies that demonstrated significant increases to biochemical and functional properties of self-assembled constructs [22], [47] and constructs formed with other methods [35], [43], [48]–[53]. On days in which constructs were to be stimulated, empty platens were placed in the stimulation device for calibration and zero position measurement. Following this, constructs were loaded into the bottom platen and the top platen was placed on top of temporary spacers in the bottom platen to prevent construct crushing. The platens were loaded into the stimulator, temporary spacers were removed, and the height of the constructs was determined by moving the top platen downwards until a force of 0.2 N was obtained. The height was inputted into the computer controlling the stimulator, enabling compression at 10% strain and 1 Hz to proceed. Each of the experimental groups designated for mechanical stimulation were loaded one at a time into the bioreactor. For example, the 5 constructs designated to the CND1 group were placed onto the compression platens and subjected to mechanical stimulation for 1 hr. These constructs were then removed and replaced with the 5 constructs from the CYD1 group which then underwent mechanical stimulation.

### Construct Processing

**Figure 2:**
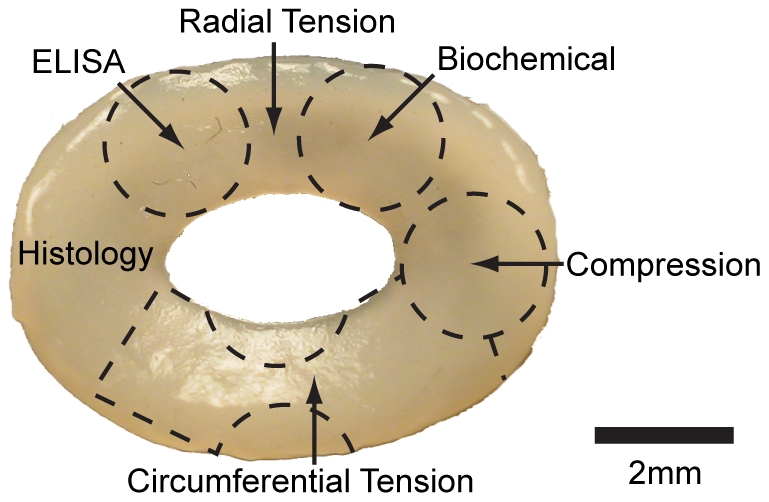
Construct division for histological, biochemical, ELISA, and biomechanical assessments. For biochemical, ELISA, and compressive testing 2 mm punches were removed from the construct. Tensile specimens were prepared by fashioning dumbbell shaped portions of the construct in the appropriate direction for circumferential and radial testing. After obtaining the above specimens, a sufficient portion was available for histological assessment.

At the end of the 4 wk culture period construct wet weight and gross morphological images were obtained. Constructs were then divided as shown in Figure 2 to obtain samples for biochemical, biomechanical, and histological assessments. From two constructs in each experimental group, samples were taken for histological examination in both the circumferential and radial oriented directions. For all other assessments, 5 samples from each experimental group were used.

### Histology

Orientation in either the circumferential or radial direction was noted when samples were snap-frozen at −20°C in HistoPrep™ (Fisher Scientific). Sections 14 µm thick were stained with picrosirius red and safranin O/fast green for collagen and GAG distribution visualization, respectively. Picrosirius red stained sections were viewed under polarized light to visualize collagen fibril orientation. Immunohistochemistry for collagen I and collagen II was performed by using protocols for the Vectastain ABC and DAB Substrate kit (Vector Laboratories) in conjunction with anti-collagen I (Axell) and anti-collagen II antibodies (Cedar Lane Laboratories).

### Biochemistry

Wet and dry weights of biochemical samples were taken. Samples were then digested in a 125 µg/mL papain (Sigma) for 18 hrs at 65°C. A modified hydroxyproline assay was used to determine collagen content [54]. The Blyscan (Biocolor) assay kit was used to quantify GAG. The PicoGreen dsDNA reagent (Invitrogen) was used to quantify DNA amount and a conversion factor of 7.7 pg DNA/cell was used to convert to cell number.

### ELISA

Construct samples were digested via sequential pepsin and elastase treatments and then processed for collagen I and II quantification. For the collagen II ELISA, Chondrex reagents and protocols were used. For the collagen I ELISA, a similar protocol was employed with antibodies from US Biological. Briefly, these protocols were sandwich ELISAs in which a capture antibody was first allowed to adsorb onto an ELISA plate, followed sequentially by BSA for blocking, samples and standards, detection antibody, peroxidase linked complex, TMB, and HCl.

### Compression Testing

Prior to construct testing, compression samples were photographed and sample diameter was measured using ImageJ. The height of the sample was determined by moving the platens of an EnduraTEC ELF 3200 system (BOSE-Electroforce) into contact, zeroing the displacement, placing the sample onto the lower platen, and then slowly lowering the upper platen until a load of 0.2N was reached while measuring platen to platen separation. Construct compressive properties were assessed using unconfined, stepwise stress relaxation testing. Samples were placed in a PBS bath and, while the force data were recorded, compressed to 10% and 20% strain with a 10 minute relaxation period following both strain levels. There data were analyzed with a custom program and the MatLab curve fitting toolbox (Math Works) to determine the viscoelastic properties (relaxation modulus, instantaneous modulus, and coefficient of viscosity) as described previously [55].

### Tension Testing

Dumbbell-shaped tensile samples were photographed to determine thickness and width using ImageJ. Tensile samples were adhered with cyanoacrylate glue to the strips of paper cut with a consistent gap to standardize gauge length, secured into the grips of an Instron 3340, and the paper was cut so that only the constructs would be subjected to tension. While measuring grip to grip displacement, the constructs were strained at 1% of the gauge length per second until failure. The test data was loaded into Matlab, the linear region of the curve was isolated via a custom program, and the Young's modulus was determined.

### Statistical Analysis

Each group consisted of *n = 5* for biochemical, compression, and tensile testing. Results of these tests were analyzed with a two-factor ANOVA. When the main effects test showed significance (p < 0.05), Tukey's HSD post hoc test was performed to determine significant differences among the levels of a particular factor or among all groups. Also, the interaction term obtained from the two-factor ANOVA involving the four groups of interest was used to assess synergy between treatments with p < 0.05 defined as significant [56]. In subsequent Figures illustrating geometric, biochemical, and biomechanical data, statistical significance between levels of a given factor or individual groups is present when a letter is not shared. For comparison of circumferential and radial tensile moduli a paired t-test was used to determine if the direction of testing significantly (p < 0.05) altered the tensile modulus.

## Results

### Gross Morphology

**Figure 3:**
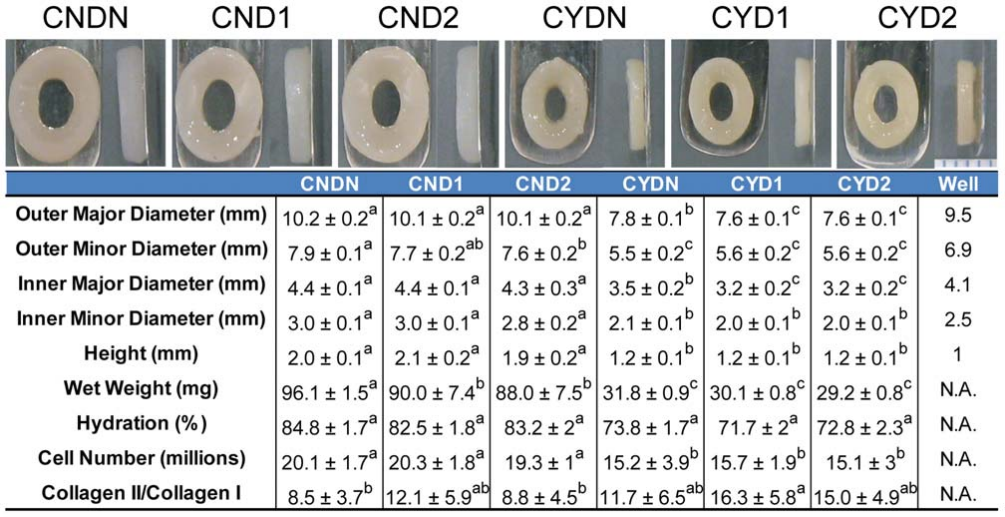
Construct gross morphological images and properties. White scale bar in lower right corner of gross morphology images is equal to 5 mm. Geometric properties, construct wet weight, hydration, cell number, and Collagen II/Collagen I ratio are shown in tablature form and illustrate the large effect on these properties by chemical stimulation. For group labeling, the letter following the C denotes if chemical stimulation was applied (N = no, Y = yes) and the character following the D denotes the time window of mechanical stimulation application (N = none, 1 = days 10–14, 2 = days 17–21). All data are presented as mean ± s.d. Statistically significant differences are present between values that do not share a common letter for a particular metric.

All constructs were able to withstand the level of mechanical stimulation without tearing or permanently deforming. Geometric properties, wet weight, hydration, and gross morphological images after 4 wks of culture and geometric properties of the self-assembly well are displayed in Figure 3. Via a two-way ANOVA, chemical stimulation was found to significantly decrease all geometric properties, wet weight, and hydration. Mechanical stimulation did not significantly affect geometric properties, except the major axis diameter, but did significantly lower the hydration of the early mechanical stimulation group and the WW of the late mechanical stimulation group. Wet weights ranged from 29 to 96 mg and hydration ranged from 72 to 85%. Examination of gross morphological images highlights the differences in morphological properties but also shows, more apparently in side view images, a difference in construct coloration. The darker hue of these constructs is likely indicative of enhanced ECM density.

### Histology and Immunohistochemistry

**Figure 4:**
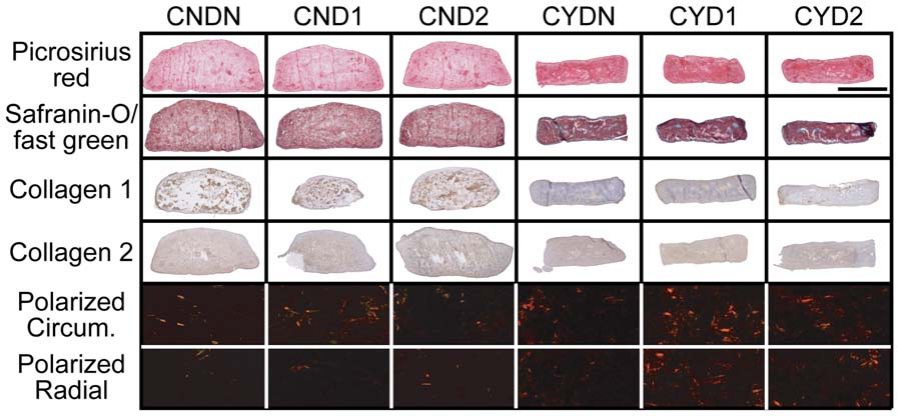
Histological staining. (Row 1) Picrosirius red staining for collagen content. (Row 2) Safranin = O/fast green staining for GAG content. (Row 3) Collagen 1 IHC. (Row 4) Collagen 2 IHC. (Row 5) Polarized light imaging of picrosirius red staining in circumferential direction. (Row 6) Polarized light imaging of picrosirius red rtaining in radial direction. Polarized light images are taken such that fiber orientation in the relevant direction will be horizontal in all images. The length of the black bar in the upper right corner represents 2 mm for all images except polarized light which were taken at 5x.

Images from the histological assessment for collagen and GAG and the immunohistological assessment for collagen I and collagen II are found in Figure 4. Qualitatively, picrosirius red stain intensity exhibits an obvious increase when chemical stimulation is applied. Visualization of picrosirius red stained sections with polarized light revealed a qualitative increase in birefringence intensity and frequency with circumferential orientation as compared to radial orientation. A greater amount of birefringence was also noted in chemically stimulated samples. Safranin O/fast green stain intensity also qualitatively increased with chemical stimulation but no readily apparent differences were observed due to mechanical stimulation. Collagen I staining confirms the presence of this protein along the periphery of chemically stimulated constructs and throughout the non-chemically treated constructs. Collagen II staining reveals consistent intense staining in all groups.

### Biochemistry

**Figure 5:**
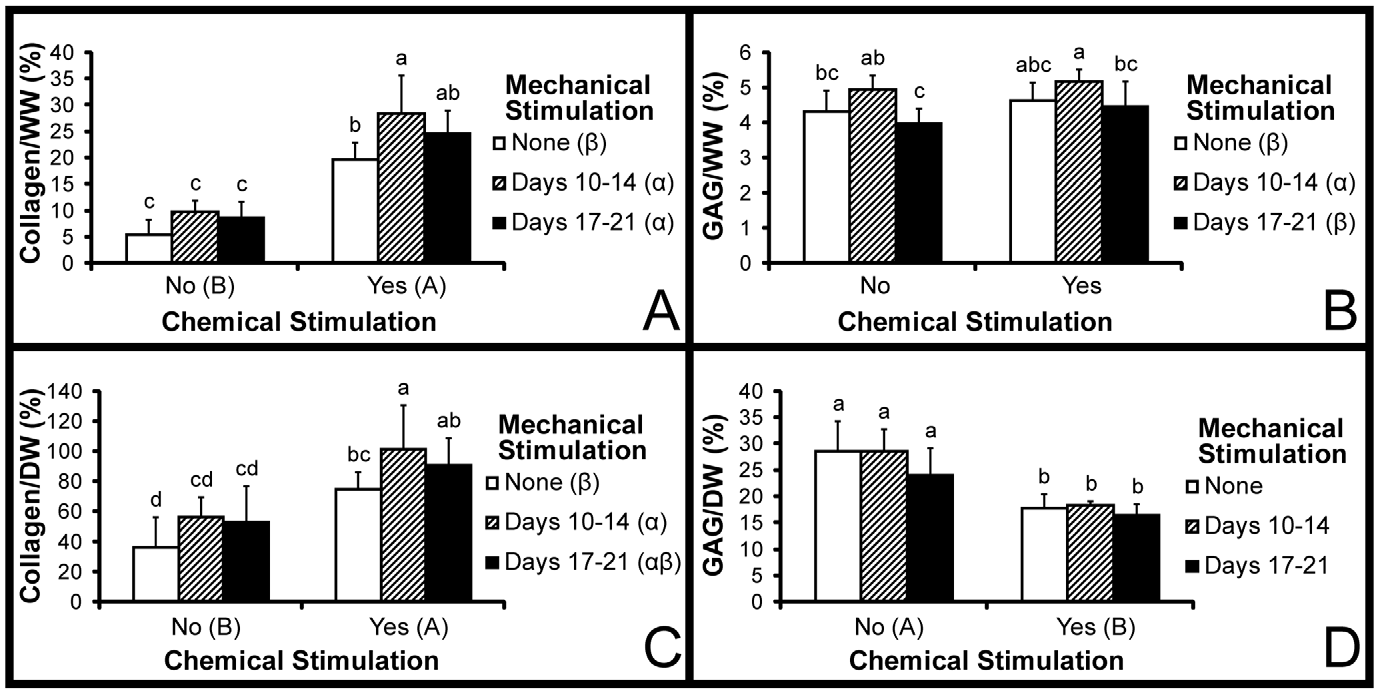
Biochemical properties. Both collagen per wet weight (A) and collagen per dry weight (C) were significantly increased by chemical and mechanical stimulation. GAG per wet weight (B) was significantly increased by D1 treatment only while GAG per dry weight (D) was decreased by chemical stimulation. All data are presented as mean ± s.d. Statistically significant differences are present when a common letter is not shared between levels of a factor or by individual groups. For mechanical stimulation, the characters α, β, and γ are used. For chemical stimulation, letters A and B are used. For comparisons among the 6 treatment groups, the letters a, b, c, d, and e are used.

Biochemical tests for collagen and GAG were normalized to both wet weight (WW) and dry weight (DW) and shown in Figure 5. Results of biochemical tests used to quantify the number of cells per constructs and the collagen II to collagen I ratio are shown in Figure 3. Collagen/WW ranged from 5–28% with a statistically significant 4-fold additive increase over CNDN associated with CYD1 treatment. A two-way ANOVA showed that chemical stimulation and both regimens of mechanical stimulation significantly enhanced Collagen/WW. These significant differences were maintained in Collagen/DW where a 1.8-fold increase over CNDN resulted from CYD1 treatment. GAG/WW ranged from 4–5% with a statistically significant increase observed with early mechanical stimulation but no change as a result of chemical stimulation. Conversely, chemical stimulation resulted in a statistically significant decrease in GAG/DW but was not affected by mechanical stimulation. The collagen II to collagen I ratio showed an abundance of collagen II with values ranging from 8.5 to 16.3 with a statistically significant increase associated with chemical stimulation. Although not significant, mechanical stimulation, particularly early stimulation, trended towards increasing this metric as well. Non-chemically stimulated constructs possessed the same number of cells that had been seeded, while chemical stimulation resulted in a 25% decrease in cell number.

### Biomechanics

**Figure 6:**
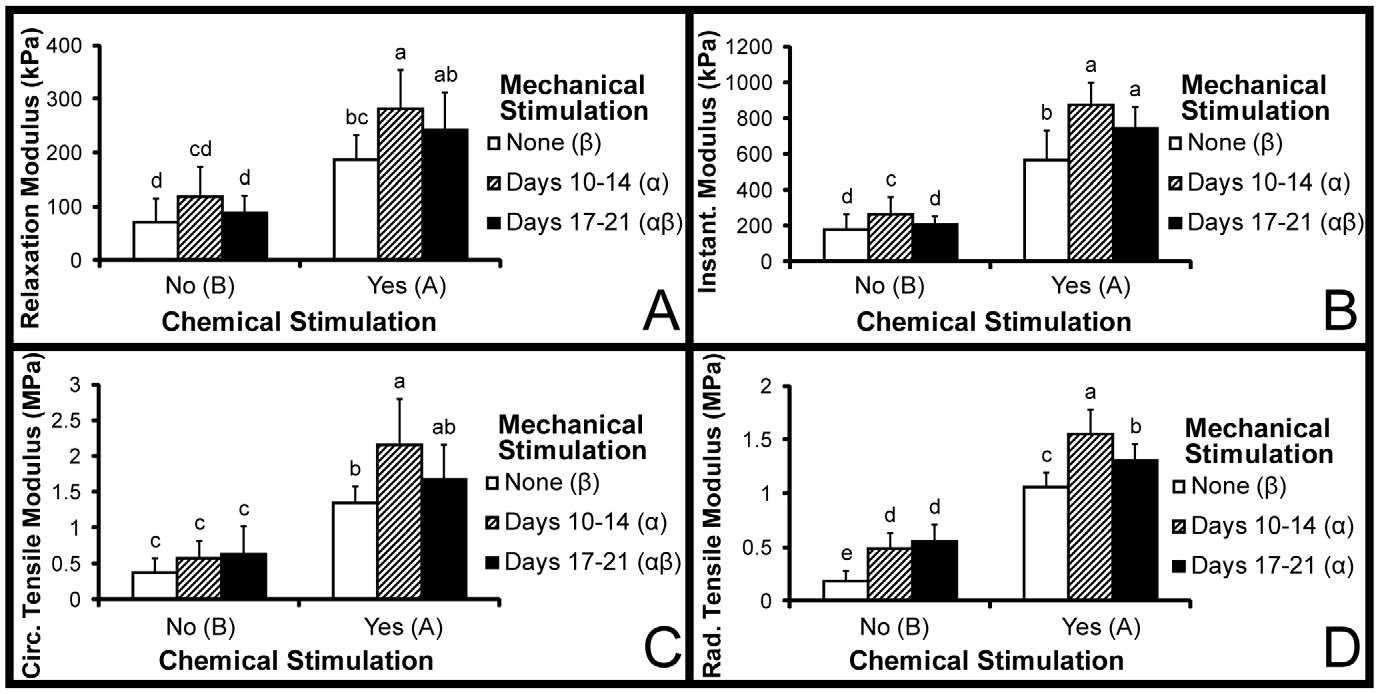
Biomechanical properties. (A) compressive relaxation modulus at 10% strain (B) compressive instantaneous modulus at 10% strain (C) circumferential Young's tensile modulus (D) radial Young's tensile modulus. All mechanical properties were significantly enhanced by chemical stimulation and D1 mechanical stimulation. All data are presented as mean ± s.d. Statistically significant differences are present when a common letter is not shared between levels of a factor or by individual groups. For mechanical stimulation, the characters α and β are used. For chemical stimulation, letters A and B are used. For comparisons among the 6 treatment groups, the letters a, b, c, d, and e are used.

Results of the compressive and tensile biomechanical assessments are found in Figure 6. Two-way ANOVAs showed statistically significant increases in all construct functional properties over their corresponding no-treatment control due to chemical stimulation and early mechanical stimulation. While late mechanical stimulation trended towards increasing many of the functional properties, the only statistically significant increase as a result of this stimulation was the radial tensile modulus. In terms of compressive properties at 10% strain, the relaxation modulus ranged from 71 to 281 kPa with a statistically significant 2-fold increase over CNDN obtained by CYD1 treatment. The instantaneous modulus ranged from 176 to 872 kPa with a statistically significant 3-fold increase over CNDN obtained by CYD1 treatment. As the same statistical trends observed at 10% strain were found at 20% strain, these data are not shown. Young's tensile moduli in the circumferential direction ranged from 0.4 to 2.2 MPa with a statistically significant 4-fold increase over the CNDN group resulting from CYD1 treatment. Young's tensile moduli in the radial direction ranged from 0.2 to 1.5 MPa with a statistically significant 6-fold increase over CNDN obtained with CYD1 treatment. The direction of tensile testing resulted in a statistically significant decrease in Young's tensile modulus in the radial direction as compared to the circumferential direction. Considering the two groups that received chemical stimulation and either early or late mechanical stimulation, the only significant difference in functional properties was the tensile modulus in the radial direction. However, CYD1 stimulation significantly enhanced all functional properties over those of the CYDN group; whereas CYD2 treatment only significantly increased the radial tensile modulus over the CYDN group.

## Discussion

This study significantly advances meniscus tissue engineering by being the first to 1) apply simultaneous tension-compression mechanical stimulation to an anatomically-shaped meniscus construct, 2) observe enhancement in tensile and compressive properties of a meniscus construct in response to mechanical stimulation, and 3) apply combined C-ABC and mechanical stimulation to cartilaginous constructs. The central hypothesis of this study was that by combining chemical and mechanical stimulation additive increases to construct biochemical and biomechanical properties would be obtained. This was statistically proven for all biochemical and functional properties as evidenced by the level of increase observed following combined mechanical and chemical stimulation in comparison to the level of enhancement due to single application of either chemical or mechanical stimulation. The secondary hypothesis regarding the benefit earlier mechanical stimulation was also proven through significant increases in collagen/WW, and compressive and tensile moduli due to this treatment. Overall, this study shows that construct functional properties can be enhanced through both chemical or tension-compression mechanical stimulation and that these increases are additive when these stimuli are applied in concert.

The results of mechanical stimulation found in this study are in agreement with previous research that has demonstrated increases in the biochemical and biomechanical properties of engineered menisci in response to either compressive or tensile mechanical stimulation. Application of dynamic compression to meniscus cell seeded agarose gels resulted in improvements in proline and sulfate incorporation compared to statically compressed controls [39]. In response to compressive stimulation of meniscus cell-seeded alginate gels, Ballyns [41] observed significant increases in collagen/WW, GAG/WW, and equilibrium modulus. With regards to dynamic tension, Vanderploeg et al. [38] reported no changes to cell seeded fibrin constructs, Upton et al. [40] identified an increase in proline incorporation, and Baker et al. [19] noted increases in collagen, GAG, and stiffness with cells from some donors. Compared to non-mechanically stimulated controls, the present study determined that mechanical stimulation increased collagen per wet weight (up to 80%), GAG per wet weight (up to 14%), relaxation modulus (up to 66%), instantaneous modulus (up to 54%), circumferential tensile modulus (up to 65%), and radial tensile modulus (up to 200%). The results presented in this study significantly enhance the field of meniscal tissue engineering by demonstrating improvement of all major functional and biochemical properties following simultaneous tension-compression stimulation and, for the first time, show that mechanical stimulation is beneficial to scaffold-free meniscal constructs.

Two distinct growth phenotypes have been described for cartilaginous tissues: appositional and maturational [27]. Appositional growth is characterized by increased tissue size and wet weight and decreased collagen per wet weight, GAG per wet weight, and tensile properties [27]. This growth phenotype has been described during *in vitro* culture of cartilage explants and is due to an imbalance between GAG and collagen production, an imbalance which results in tissue swelling and loss of tensile properties [27]. Maturational growth occurs when matrix is concentrated within the tissue as evidenced by increased collagen per wet weight, GAG per wet weight, and tensile properties, with concomitantly decreased hydration [27]. The biochemical and biomechanical results of this study demonstrate maturational growth of engineered meniscal constructs in response to C-ABC and TGF-β1 treatment and are in agreement with previous studies that report the same finding [11], [18], [27], [28]. TGF-β1 has been shown to not only increase collagen synthesis but also α-SMA expression [11]. α-SMA enhances the contractile nature of cartilaginous cells allowing concentration of the ECM components within the construct. C-ABC eliminates GAGs from the construct and, thus, GAG-associated swelling due to the ability of GAGs to attract water. The reduction of swelling pressure causes the pre-stressed collagen matrix to collapse onto itself, potentially allowing additional cell-cell, cell-collagen, and collagen-collagen interactions to occur [11], [18], [27], [28]. In addition, the increased collagen per wet weight and tensile properties suggest maturational growth in response to mechanical stimulation. Furthermore, by eliminating the pre-stress associated with GAGs the effect of the mechanical stimulation may be amplified, thus, resulting in additive increases to biochemical and biomechanical properties. These two chemical stimuli work in concert with mechanical stimulation to concentrate ECM within the construct, promoting maturational growth and increased functional properties.

One of the most significant results of this study is the similarities between the engineered menisci and native menisci. Although, engineered menisci exposed to the CYD1 treatment were smaller than the well into which they were seeded, their size would still be appropriate for leporine inner-meniscus replacement. Also, these constructs were the correct height and possessed the curved-wedge profile that is critical for proper load transmission. Collagen/WW obtained for the CYD1 treatment (28%) is on par with native tissue (22%) [3]. Furthermore, the prevalence of collagen orientation in the circumferential direction is similar to the direction of collagen orientation in native menisci [3]. The GAG/WW value associated with this treatment (5%) compares well to native tissue (3–5%) [3]. The compressive relaxation modulus of meniscal constructs at 10% strain (281 kPa) exceeds that of native tissue at 12% strain (137 kPa) [57] and the instantaneous modulus (871 kPa) is on par with native tissue (1130 kPa) [57]. While tensile properties of meniscal constructs in the radial direction (1.5 MPa) approximate native tissue (3 MPa) [58], tensile properties in the circumferential direction (2.1 MPa) need to be addressed to match values of native menisci (160 MPa) [58]. These favorable comparisons to native tissue are encouraging because previously it has been exceedingly challenging to mirror the functional properties and collagen content of native menisci.

This study shows for the first time the significant benefits of combining TGF-β1, C-ABC, and simultaneous tension-compression stimulation and in the process opens many new paths of investigation. The main focus of future studies should be on further enhancing matrix organization and collagen maturation. Optimization of the well confinement time has been shown to aid in collagen organization [59]. This would be particularly beneficial to meniscus-shaped constructs because it is believed that the passive stresses imparted by the well result in the observed circumferential organization [7], [8]. Most importantly, longer duration tension-compression stimulation must be performed to provide the hoop strains necessary for collagen alignment and, potentially, maturation. This could include investigating stimulation for longer than 1 hr per day, increasing the number of total days of stimulation, or increasing the total culture time past 4 wks. Also, this study did not address optimization of the frequency, strain percentage, or strain rate of mechanical stimulation. Future studies should address these parameters as well. Experiments where magnitudes of the tension or compression loading can be manipulated independently, then combined, will allow for the examination of several tension magnitudes over one compression magnitude, and vice versa. Such experiments will better elucidate how the tension-compression loading is mechanotransduced to result in the currently observed increases in construct biochemical and mechanical properties.

Combined mechanical and chemical stimulation resulted in additive increases in biochemical and biomechanical properties suggesting that the mechanisms by which these increases are obtained are distinct. As discussed, maturational growth is a major factor contributing to the benefits of the chemical stimulation employed in this study. Mechanical stimulation was able to further this maturational growth by enhancing matrix content and decreasing hydration while not increasing construct size. The result of appropriately timing mechanical stimulation to chemical stimulation was the generation of engineered menisci that approximated the geometric, biochemical, and biomechanical properties of the inner portion of the rabbit meniscus.
